# Supplementary material for: Detecting Environmental Stress In Situ Using Molecular Data: A Case Study with the Filamentous Green Alga Klebsormidium and Antarctic Biocrusts
Source: Microorganisms. 2025 Sep 9;13(9):2108. doi: 10.3390/microorganisms13092108 (PMC12472361; doi:10.3390/microorganisms13092108)
Supplement: Supplementary file 1 [file microorganisms-13-02108-s001.zip › microorganisms-3748436-supplementary.pdf]

Supplementary Table 1. Characteristics of the novel assembled stress transcriptomes of K. flaccidum and K. dissectum.

		K. flaccidum	***K. dissectum***
Raw reads	Bases before processing	1,105,905,918	926,908,585
	Bases after processing	236,866,678	172,794,626
Summary of de novo assembly	Transcripts	120,574	96,581
	Genes	72,129	56,082
	N50 [bases]	3,687	3,219
Summary of BUSCO analysis	Complete (single - copy)	8%	13.18%
	Complete (duplicated)	88%	82.82%
	Fragmented	2.82%	3.29%
	Missing	1.18%	0.71%
Summary of ORF detection	Complete	78.06%	81.85%
	5′-Partial	8.83%	7.85%
	3′-Partial	6.08%	5.34%
	Internally	7.03%	4.96%
Transcripts with Blast hits		71,649	73,763
Transcripts with InterProScan hits		71,648	73,763

Supplementary Table 2. List of annotated transcripts used for GMIs from site 1 and site 3. The number of combinations the GMI was within the range from 0 to 100% is also given.

Site 1		
**Effect of Cold**		
	**Genes**	**Number of combinations in range**
Constant expression	GAQ86045.1 NDH subcomplex B4	3
	GAQ87359.1 Hypothetical protein KFL_003480030	4
	GAQ89524.1 Mitochondrial F1F0-ATP synthase subunit delta/ATP16	2
	GAQ87189.1 RRM superfamily protein	3
	GAQ77625.1 20S proteasome regulatory subunit beta protein	4
	GAQ78107.1 hypothetical protein KFL_000080110	3
	GAQ88143.1 mitochondrial or chloroplast ribosomal protein L18 precursor	3
	GAQ91160.1 hypothetical protein KFL_007360090	4
	GAQ87153.1 hypothetical protein KFL_003350030	3
	GAQ85539.1 chloroplast ribosomal protein L15 precursor	3
	GAQ80855.1 GLYCINE DECARBOXYLASE COMPLEX H	3
	GAQ90597.1 hypothetical protein KFL_006600020	3
	GAQ79238.1 glutathione peroxidase	2
Regulated genes	GAQ92806.1 chloroplast 50S ribosomal protein L23, precursor	3
	GAQ81886.1 ribulose-bisphosphate carboxylase small chain	6
	GAQ89115.1 hypothetical protein KFL_004880100	2
	GAQ84785.1 Ribosomal protein L7Ae/L30e/S12e/Gadd45 family protein	5
	GAQ79801.1 phosphoglycerate kinase	2
	GAQ81521.1 Mitochondrial or chloroplast ribosomal protein L5 precursor	2
	GAQ84878.1 DEAD/DEAH box RNA helicase family protein	4
	GAQ89501.1 hypothetical protein KFL_005300050	5

	GAQ89577.1hypothetical protein KFL_005380030	6
	GAQ91064.1 hypothetical protein KFL_007220030	3
	GAQ90219.1 hypothetical protein KFL_006150010	2
Effect of desiccation		
Constant expression	GAQ86045.1 NDH subcomplex B4	1
	GAQ87359.1 Hypothetical protein KFL_003480030	1
	GAQ89524.1 Mitochondrial F1F0-ATP synthase subunit delta/ATP16	1
	GAQ86081.1 hypothetical protein KFL_002690070	2
	GAQ78107.1 hypothetical protein KFL_000080110	1
	GAQ88143.1 mitochondrial or chloroplast ribosomal protein L18 precursor	1
	GAQ91160.1 hypothetical protein KFL_007360090	2
	GAQ85539.1 chloroplast ribosomal protein L15 precursor	1
	GAQ80855.1 glycine decarboxylase complex H	4
	GAQ79238.1 glutathione peroxidase	1
	GAQ85911.1 Malate dehydrogenase	1
	GAQ89281.1 Mitochondrial F1F0-ATP synthase subunit epsilon/ATP15	1
Regulated genes	GAQ92806.1 chloroplast 50S ribosomal protein L23, precursor	1
	GAQ92391.1 Chloroplast protein	1
	GAQ86140.1 LysM domain containing protein	3
	GAQ84471.1 cytoplasmic glutamyl-tRNA synthetase	2
	GAQ82440.1 hypothetical protein KFL_001110160	1
	GAQ82929.1 hypothetical protein KFL_001290200	1
	GAQ82955.1 reticulon domain containing protein	5
	GAQ85931.1 hypothetical protein KFL_002610170	3

**Site 3**		
**Effect of Cold**		
	**Genes**	**Number of combinations in range**

Constant expression	GAQ80845.11-cysteine peroxiredoxin 1	1
	GAQ82617.1F0F1-type ATP synthase gamma subunit	1
	GAQ87971.1phosphoribulokinase	2
	GAQ89666.1chloroplast import apparatus	5
	GAQ88720.1LysM domain containing protein	5
	GAQ90049.1hypothetical protein KFL_005930060	6
	GAQ82317.1glutamate--glyoxylate aminotransferase	2
	GAQ86930.1hypothetical protein KFL_003210060	6
	GAQ90049.1hypothetical protein KFL_005930060	5
	GAQ88311.1Histones H3 and H4	1
	GAQ87005.1putative pyridoxine biosynthesis protein	5
	GAQ83893.1pyrophosphorylase	5
	GAQ88602.1AWPM-19-like family protein	5
Regulated genes	GAQ81872.1chloroplast ribosomal protein S20 precursor	7
	GAQ82344.1ascorbate peroxidase	8
	GAQ86998.1autophagy-related protein 8	1
	GAQ92806.1chloroplast 50S ribosomal protein L23, precursor	2
	GAQ83104.1allene oxide cyclase	8
	GAQ79986.1hypothetical protein KFL_000430280	8
	GAQ85520.1photosystem II oxygen-evolving enhancer protein II-1	1
	GAQ81646.1Ankyrin repeat domain-containing protein 2	4
	GAQ78745.1hypothetical protein KFL_000180280	8
	GAQ80541.1S-adenosylhomocysteine hydrolase	2
Effect of desiccation		
Constant expression	GAQ80845.11-cysteine peroxiredoxin 1	1
	GAQ87971.1phosphoribulokinase	2
	GAQ89666.1chloroplast import apparatus	3
	GAQ88720.1LysM domain containing protein	4
	GAQ90049.1hypothetical protein KFL_005930060	4
	GAQ82317.1glutamate--glyoxylate aminotransferase	1
	GAQ86930.1hypothetical protein KFL_003210060	5
	GAQ90049.1hypothetical protein KFL_005930060	4
	GAQ88311.1Histones H3 and H4	1

	GAQ87005.1putative pyridoxine biosynthesis protein	5
	GAQ83893.1pyrophosphorylase	5
	GAQ88602.1AWPM-19-like family protein	5
Regulated genes	GAQ80571.1photosystem I subunit IV	4
	GAQ86945.1Protein kinase-like superfamily protein	8
	GAQ91027.1pyruvate dehydrogenase E1 component subunit beta	1
	GAQ77944.1hypothetical protein KFL_000060090	3
	GAQ90257.1hypothetical protein KFL_006190070	8
	GAQ84403.1hypothetical protein KFL_001870250	1
	GAQ84700.1alpha beta-hydrolases superfamily protein	9
	GAQ82955.1reticulon domain containing protein	6
